# Three-Dimensional Flow of Nanofluid Induced by an Exponentially Stretching Sheet: An Application to Solar Energy

**DOI:** 10.1371/journal.pone.0116603

**Published:** 2015-03-18

**Authors:** Junaid Ahmad Khan, M. Mustafa, T. Hayat, M. Sheikholeslami, A. Alsaedi

**Affiliations:** 1 Research Centre for Modeling and Simulation (RCMS), National University of Sciences and Technology (NUST), Islamabad 44000, Pakistan; 2 School of Natural Sciences (SNS), National University of Sciences and Technology (NUST), Islamabad 44000, Pakistan; 3 Department of Mathematics, Quaid-I-Azam University 45320, Islamabad 44000, Pakistan; 4 Department of Mathematics, Faculty of Science, King Abdulaziz University, P. O. Box 80257, Jeddah 21589, Saudi Arabia; 5 Department of Mechanical Engineering, Babol University of Technology, Babol, Iran; North China Electric Power University, CHINA

## Abstract

This work deals with the three-dimensional flow of nanofluid over a bi-directional exponentially stretching sheet. The effects of Brownian motion and thermophoretic diffusion of nanoparticles are considered in the mathematical model. The temperature and nanoparticle volume fraction at the sheet are also distributed exponentially. Local similarity solutions are obtained by an implicit finite difference scheme known as Keller-box method. The results are compared with the existing studies in some limiting cases and found in good agreement. The results reveal the existence of interesting Sparrow-Gregg-type hills for temperature distribution corresponding to some range of parametric values.

## Introduction

Boundary layer flow due to an impulsive motion of a moving extensible surface is involved in various industrial and technological applications such as metal and polymer extrusion, aerodynamic extrusion of plastic sheets, glass blowing, crystal growing, paper production etc. Sakiadis [1] has done the pioneering work on the boundary layer flow over a moving plate in a stationary ambient fluid. Crane [2] studied the Sakiadis problem for a stretching sheet and obtained a closed form exact solution for the velocity distribution. Rajagopal et al. [3] considered the flow of elasto—viscous fluid boundedby a stretching sheet. He concluded that rate of cooling of the extruded polymer sheet is larger in viscoelastic fluid when compared with the viscous fluid. Lawrence and Rao [4] extended this work for heat transfer characteristics. After these fundamental studies, stretching sheet problem in two- and three-dimensional flows has been extensively studied by the researchers (see Grubka and Bobba [5], Banks [6], Chen and Char [7], Ali [8], Pop and Na [9], Magyari and Keller [10],Liao [11,12], Xu et al. [13], Sajid et al. [14], Liu and Anderson [15], Xu and Liao [16], Hayat et al. [17] and Junaid et al. [18]). These studies were only confined to the flow over linearly stretching surfaces. However, in industrial applications mentioned above, the velocity of the extruded sheet may not be necessarily linear. Keeping this in view, Magyari and Keller [19] considered the two-dimensional viscous flow over caused by an exponentially stretching sheet. In this work, the surface heat transfer was also exponentially distributed. Khan and Sanjayanand [20] examined heat transfer of viscoelastic boundary layer flow over an exponentially stretching sheet and obtained an approximate analytical solution. Homotopy analytic solutions for two-dimensional flow over an exponentially stretching sheet with thermal radiation were presented by Sajid and Hayat [21]. Radiation effects on the boundary layer flow of Jeffrey fluid above an exponentially stretching sheet were described by Nadeem et al. [22]. Recently Liu et al. [23] provided an excellent numerical study on the three-dimensional viscous flow past an exponentially stretching sheet.

Traditional heat transfer fluids such as water, ethylene-glycol, engine oil, lubricants etc. possess limited heat transfer capabilities due to their low thermal conductivity and are inadequate to meet the modern cooling requirements. On the other hand metals possessextremely higher thermal conductivity in contrast to the conventional heat transfer fluids. Masuda et al. [24] initially pointed out that viscosity and thermal conductivity of the liquids can be altered by using nanoparticles (usually made up of metals, oxides, carbides andcarbon nanotubes) in the base fluids. Choi and Eastman [25] have observed the unexpected increase in the thermal conductivity through the dispersion of nanoparticles in the base fluid. The enhanced thermal behavior of nanofluids has vital importance in many industrial fields including power generation, transportation, micro-manufacturing, micro-electronics, pharmaceutical processes,thermal therapy for cancer treatment, chemical and metallurgical sectors, air-conditioning etc. In automobiles, the application of nanofluids as coolants would allow for better size and positioning of the radiators and hence this will require less energy for overcoming resistance on the road. Due to a significant improvement in vehicle aerodynamics, there is higher demand for braking systems with higher and more efficient heat dissipation mechanisms and properties such as brake nanofluid. Researchers also suggested the use nanofluid based solar collectors for optimal absorption of solar radiations (see Trieb and Nitsch [26], Otanicar et al. [27] and Ladjevardi et al. [28]). The magnetic nanoparticles are important in medicine, construction of loud speakers, sink float separation, cancer therapy and tumor analysis. The thermal properties of magnetic nanoparticles are also tunable through the variations in the magnetic field strength. It is also pointed out recently that magnetic nanoparticles are injected into the blood vessels nearest to the cancerous tissues [29].

In view of the above mentioned applications, Buongiorno [30] studied the convective transport in nanofluids and concluded the Brownian motion and thermophoresis as the most important mechanisms for the abnormal heat transfer enhancement. Natural convective boundary layer flows of nanofluids past a vertical flat plate were explored by Kuznetsov and Nield [31] and Nield and Kuznetsov [32]. They derived the governing equations for nanofluid flow through Buongiorno’s model. It is also evident that rate of cooling of the extruded polymer sheet can be improved by using nanofluids. In this regard the classical problem of two-dimensional flow over alinearly stretching sheet in the presence of nanoparticles was conducted by Khan and Pop [33]. LaterMakinde and Aziz [34] revisited the work of Khan and Pop [33] by considering convective boundary condition. Mustafa et al. [35] provided analytic solution for stagnation-point flow of a nanofluid by using homotopy analysis method (HAM). Mustafa et al. [36, 37] used HAM to explore the two-dimensional exponentially stretching sheet problem for nanofluids. Rana and Bhargava [38] discussed the flow of nanofluid over a nonlinearly stretching sheet by finite element method. Bég et al. [39] numerically investigated the unsteady MHD mixed convective boundary layer flow of a nanofluid induced byan exponentially stretching sheet embedded in a porous medium. Numerical solution for nanofluid flow past a stretching cylinder with non-uniform heat source was considered by Rasekh et al. [40]. Uddin et al. [41] discussed thesteady two-dimensional MHD free convective boundary layer flow of an electrically conducting nanofluid past a vertical flat plate with Newtonian heating boundary condition. Ashorynejad et al. [42] investigated nanofluid flow over stretching cylinder in the presence of magnetic field. Mustafa et al. [43] examined the unsteady boundary layer flow of nanofluid past an impulsively stretching sheet by HAM. Exact analytic solutions of unsteady convective heat transfer problem for various nanofluids have been derived by Turkyilmazoglu [44]. Numerical solution for non-linear radiation heat transfer problem in nanofluids with an application to solar energy was computed by Mushtaq et al. [45]. Flow of nanofluid due to a rotating disk was discussed by Turkyilmazoglu [46]. Magnetic field effects on the flow of Cu-water nanofluid were discussed by Sheikholeslami et al. [47]. Safei et. al. [48] discussed the heat transfer enhancement in nanofluids using nanotubes in forward-facing contracting channel. Malvandi and Ganji[49]examined the flow of water or aluminum based nanofluids through circular channel with magnetic field. Mixed convection flow past a vertical micro-channel was addressed by Malvandi and Ganji [50]. In another paper, Malvandi and Ganji [51] forced convection flow of nanofluid in a cooled plate micro-channel was considered. Karimipour et. al. [52]used lattice Boltzmann method to discuss the mixed convection of cu/water nanofluid inside an inclined lid driven cavity.

To the best of our knowledge the three-dimensional flow of nanofluid over an exponentially stretching sheet is not considered by the researchers. Thus currentwork is undertaken to extend the flow analysis of Liu et al. [23] for nanofluid (by incorporating the combined effects of Brownian motion and thermophoresis). Although we employ a similarity approach to non-dimensionalize the problem but since coordinates x and y could not be eliminated from the dimensionless equations, the solutions are locally similar. Such kind of solutions can be used to see the variation of parameters at fixed location above the stretching sheet (which is coincident with the *xy*—plane). Recent studies concerned with the local similarity solutions of the boundary layer equations can be found in refs. [53–59]. The equations are solvedfor the numerical solutions by Keller-box method [60, 61]. Graphs are presented to investigate the underlying physics of the problem.

## Problem Formulation

**Fig 1:**
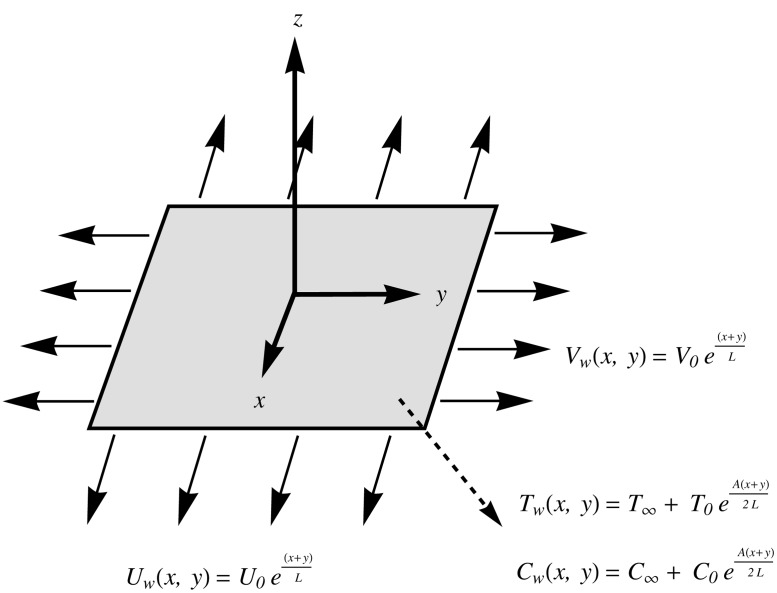
Physical configuration and coordinate system.

Consider the steady three-dimensional incompressible boundary layer flow of nanofluid over a sheet stretched exponentially in two lateral directions. The sheet is located at *z = 0* and the flow is confined to z ≥ 0. Let $U_{w}(x,y)=U_{0}e^{\frac{x+y}{L}}$ and $V_{w}(x,y)=V_{0}e^{\frac{x+y}{L}}$ be the velocities of the sheet along x—and y—directions respectively. The sheet is maintained at temperature $T_{w}(x,y)=T_{\infty }+T_{0}e^{\frac{A(x+y)}{2L}}$ while $C_{w}(x,y)=C_{\infty }+C_{0}e^{\frac{A(x+y)}{2L}}$ is the nanoparticle volume fraction at the sheet where *T*_∞_ and *C*_∞_are the ambient values of temperature and nanoparticle volume fraction respectively (see Fig. 1). Under the usual boundary layer assumptions, the equations governing the conservations of mass, momentum, energy and nanoparticles mass are(see Liu et al. [23], Kuznetsov and Nield [31], Khan and Pop [33] etc.)

$$\frac{\partial u}{\partial x}+\frac{\partial v}{\partial y}+\frac{\partial w}{\partial z}=0,$$ (1)

$$u\frac{\partial u}{\partial x}+v\frac{\partial u}{\partial y}+w\frac{\partial u}{\partial z}=v\frac{\partial^{2}u}{\partial z^{2}},$$ (2)

$$u\frac{\partial v}{\partial x}+v\frac{\partial v}{\partial y}+w\frac{\partial v}{\partial z}=v\frac{\partial^{2}v}{\partial z^{2}},$$ (3)

$$u\frac{\partial T}{\partial x}+v\frac{\partial T}{\partial y}+w\frac{\partial T}{\partial z}=\alpha \frac{\partial^{2}T}{\partial z^{2}}+\tau \left[D_{B}(\frac{\partial C}{\partial Z}\frac{\partial T}{\partial Z})+\frac{D_{T}}{T_{\infty }}{\left(\frac{\partial T}{\partial Z}\right)}^{2}\right],$$ (4)

$$u\frac{\partial C}{\partial x}+v\frac{\partial C}{\partial y}+w\frac{\partial C}{\partial z}=\left[D_{B}\left(\frac{\partial^{2}C}{\partial Z^{2}}\right)+\frac{D_{T}}{T_{\infty }}\left(\frac{\partial^{2}T}{\partial Z^{2}}\right)\right],$$ (5)

where *u*,*v* and *w* are the velocity components along the *x*-, *y*- and *z*—directions respectively, *v* is the kinematic viscosity, *T* is the fluid temperature, *C* is the nanoparticlevolume fraction, *α* is the thermal diffusivity, *D_B_* is the Brownian diffusion coefficient, *D_T_* is the thermophoretic diffusion coefficient and *τ* (= (*ρc*)_*p*_/(*ρc*)_*f*_) is the ratio of the effective heat capacity of the nanoparticle material to the effective heat capacity of the base fluid(see Table 1). The boundary conditions for the considered problem are:

$$u=U_{w}(x,y),v=V_{w}(x,y),w=0,T=T_{w}(x,y),C=C_{w}(x,y)\text{at}\;Z=0,u=0,T\to T_{\infty },C\to C_{\infty }\text{asz}\to \infty ,$$ (6)

Using the following dimensionless variables [23]

$$u=U_{0}e^{\frac{x+y}{L}}f^{\prime },\;\;v=U_{0}e^{\frac{x+y}{L}}g^{\prime },\;\;w=-\sqrt{\frac{\nu U_{0}}{2L}}e^{\frac{x+y}{2L}}\left(f+\eta f^{\prime }+g+\eta g^{\prime }\right),T=T_{\infty }+T_{0}e^{\frac{A\left(x+y\right)}{2L}}\theta ,\;C=C_{\infty }+C_{0}e^{\frac{A\left(x+y\right)}{2L}}\phi ,\;\eta =\sqrt{\frac{U_{0}}{2\nu L}}e^{\frac{x+y}{2L}}\;z,$$ (7)

Equation (1) is identically satisfied and Equations (2)−(7)take the following forms

$$f^{\prime \prime \prime }-\;\;2\left(f^{\prime }+\;g^{\prime }\right)f^{\prime }+\left(f+g\right)f^{\prime \prime }=0,$$ (8)

$$g^{\prime \prime \prime }-\;\;2\left(f^{\prime }+\;g^{'}\right)g^{\prime }+\left(f+g\right)g^{\prime \prime }=0,$$ (9)

$$\frac{1}{Pr}\theta^{''}-A\;\left(f^{\prime }+g^{\prime }\right)\theta +\left(f+g\right)\theta '+N_{b}\phi^{\prime }\theta^{\prime }+N_{t}\theta {'}^{2}=0,$$ (10)

$$\phi^{\prime \prime }-\;\text{Sc}A\;\left(f^{\prime }+g^{\prime }\right)\phi +Sc\left(f+g\right)\phi^{\prime }+\;\frac{N_{t}}{N_{b}}\theta^{\prime \prime }=0,$$ (11)

f(0)=g(0)=0,f'(0)=1,g'(0)=λ,θ(0)=1,ϕ(0)=1,f'(+∞)→0,g'(+∞)→0,θ(+∞)→0,ϕ(+∞)→0, (12)

where *λ = V*_*0*_ */U*_*0*_ is the velocity ratio, *Nb* = *τD_B_*(*C_w_* − *C*_∞_)/*v* the Brownian motion parameter, *Nt = τD*_*T*_ *(T*_*w*_ *− T*_*∞*_ *)/T*_*∞*_ *v* is the thermophoresis parameter, *Pr = v/α* is the Prandtl number, *Sc = v/D*_*B*_ is the Schmidt number. The quantities of practical interest are the skin friction coefficients *C*_*fx*_,*C*_*fy*_ and local Nusselt number *Nu*_*x*_ defined below:

$$C_{fx}=\frac{\tau_{zx}}{\frac{1}{2}\rho U_{0}^{2}},C_{fy}=\frac{\tau_{zy}}{\frac{1}{2}\rho V_{0}^{2}},Nu=\frac{xq_{w}}{k\left(T_{w}-T_{\infty }\right)},Sh=\frac{xj_{w}}{D_{B}(C_{w}-C_{\infty })},$$ (13)

where τ_*wx*_ and τ_*wy*_ are the wall shear stress along the x- and y- directions respectively, *q*_*w*_ is the wall heat flux and *j*_*w*_ is the wall mass flux. These are as under:

$$\tau_{wx}=\mu {\left(\frac{\partial u}{\partial z}\right)}_{z=0},\tau_{wy}=\mu {\left(\frac{\partial v}{\partial z}\right)}_{z=0},q_{w}=-k{\left(\frac{\partial T}{\partial z}\right)}_{z=0},j_{w}=-D_{B}{\left(\frac{\partial C}{\partial z}\right)}_{z=0}$$ (14)

Using the dimensionless variables (7), Equation (13) becomes

$$C_{fx}\sqrt{\frac{Re}{2}}e^{\frac{-3\left(x+y\right)}{2L}}=f^{\prime \prime }\left(0\right),\;\;C_{fy}\sqrt{\frac{Re}{2}}e^{\frac{-3\left(x+y\right)}{2L}}=-g^{''}\left(0\right),Nu\frac{L}{x}\sqrt{\frac{2}{Re}}e^{\frac{-\left(x+y\right)}{2L}}=-\theta^{\prime }\left(0\right)=Nur,\;\;Sh\frac{L}{x}\sqrt{\frac{2}{Re}}e^{\frac{-\left(x+y\right)}{2L}}=-\phi^{\prime }\left(0\right)=S\text{h}r,$$ (15)

where *Re = U*_*0*_ *L/v* is the local Reynolds number. The *z−* component of velocity at far field boundary can be expressed as below:

$$w\left(x,y,\infty \right)=-\frac{\nu }{L}\sqrt{\frac{Re}{2}}e^{\frac{\left(x+y\right)}{2L}}\left[f\left(\infty \right)+g\left(\infty \right)\right]$$ (16)

**Table 1:** List of symbols.

(*x*, *y*, *z*) Cartesian coordinate system	*Sh* local Sherwood number
*u*, *v*, *w* velocity components along the *x*-, *y*-, *z*- directions	*q*_*w*_ wall heat flux
*U*_*w*_, *V*_*w*_ velocity of the stretching sheet along *x*- and *y*- directions	*j*_*w*_ wall mass flux
*T* fluid temperature	*D*_*B*_ Brownian diffusion coefficient
*T*_*w*_ wall temperature	*D*_*T*_ thermophoretic diffusion coefficient
*T*_∞_ ambient fluid temperature	*Nb* Brownian motion parameter
*C* nanoparticle volume fraction	*Nt* thermophoresis parameter
*C*_*w*_ nanoparticle volume fraction at wall	’ 1st order derivative with respect to η
*C*_∞_ ambient nanoparticle volume fraction	” 2nd order derivative with respect to *η*
*U*_*0*_, *V*_*0*_, *T*_*0*_, *C*_*0*_ positive constants	‴ 3rd order derivative with respect to *η*
*L* reference length	**Greek symbols**
*A* temperature exponent parameter	*τ* ratio of effective heat capacity of the nanoparticle material to that of the base fluid
*C*_*p*_ specific heat of the nanoparticle material	*v* kinematic viscosity
*C*_*f*_ specific heat of the base fluid	*α* thermal diffusivity
Pr Prandtl number	*η* similarity variable
*SC* Schmidt number	*λ* ratio of the stretching rates
*f*, *g* dimensionless *x*- and *y*- components of velocity	*μ* dynamic viscosity
Re local Reynolds number	*ϕ* dimensionless nanoparticle volume fraction
*k* thermal conductivity	*ρ* density of the fluid
*C*_*fx*_, *C*_*fy*_ skin friction coefficient along *x*- and *y*- direction	*τ*_*wx*_, *τ*_*wy*_ wall shear stress along x- and y- direction
*Nu* local Nusselt number	*θ* dimensionless temperature

## Numerical Results and Discussion

**Fig 2:**
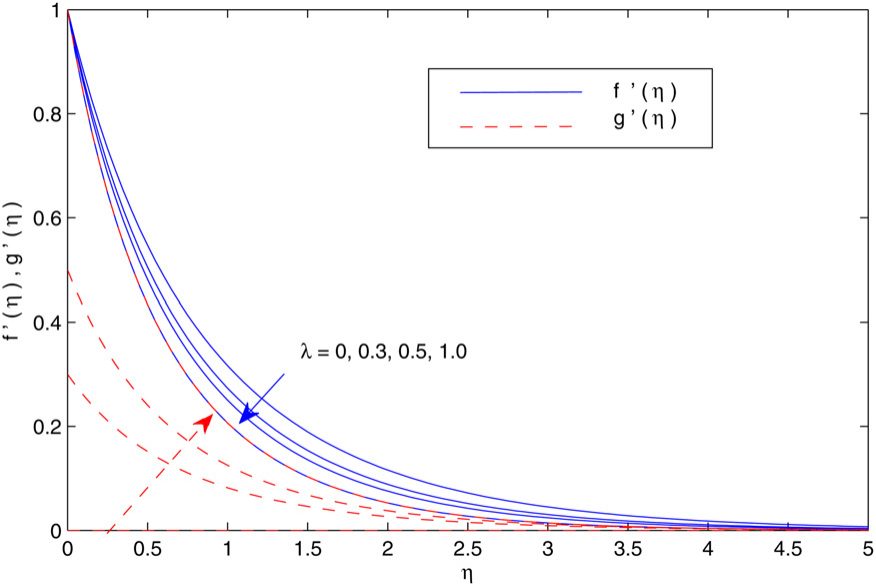
Variation in the velocity fields with *λ*.

**Fig 3:**
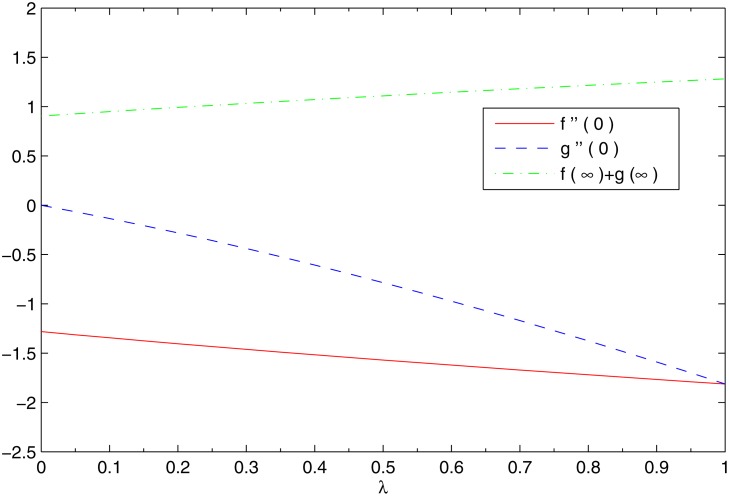
Variations in wall shear stresses and entrainment velocity with *λ*.

Keller box method has been widely applied for the solutions of boundary layer equations in fluid mechanics. This method has several attractive features such as simplicity and ease of programming, unconditional stability, second order accuracy and ability to use extrapolation as step size approaches to zero. Moreover it applies in a simple fashion to both linear and non-linear differential equations. Unlike shooting method, it can also be applied for solving non-linear partial differential equations. We solve the governing Equations (8)-(11) subject to the boundary conditions (12)by using Keller-box method. A detailed description of the method can be found in the book by Cebeci and Bradshaw [60]. The equations are transformed to first-order system by using appropriate substitutions and then reduced to difference equations using central difference. The resulting algebraic equations are linearized by using Newton’s method and written in matrix-vector form. At the end, the linear system is solved by using block-tridiagonal elimination technique. For the validation of numerical procedure, the results are compared with Magyari and Keller [19] and Liu et al. [23] inthe case of regular fluid. The numerical values are in decent agreement as can be seen from tables 2 and 3. Fig. 2 shows the profiles of dimensionless *x-* and *y-*components of velocity for different values of velocity ratio *λ*. An augmentation in *λ* indicates a larger stretching rate in the *y*-direction and thus the velocity in the *y-*direction increases whereas the velocity in the original stretching *x-*direction decreases correspondingly. In this Fig. *λ* = 0 corresponds to the two-dimensional flow case (previously reported by Mustafa et al. [36]). Fig. 3 indicates that shear stress in both the *x-*and *y-* directions increase with an increase in *λ*. As a result the entrainment velocity *f*(*∞*)+*g*(*∞*)is also an increasing function of *λ*. Thus an increase in *λ* is expected to enhance the intensity of cold ambient fluid towards the hot fluid closer to the sheet which decreases the temperature in the vicinity of the sheet.

**Table 2:** Comparison of values of wall temperature gradient ***θ’*** (0)with previous studies for the case of regular fluid *Nb = Nt = 10* ^*–5*^when *λ* = 0.

Pr	*A*	*θ’* (0)		
		Magyari and Keller [19]	Liu et al. [23]	Present (Keller-Box)
1	-1.5	0.377413	0.37741256	0.377393
	0	-0.549643	-0.54964375	-0.549641
	1	-0.954782	-0.95478270	-0.954763
	3	-1.560294	-1.56029540	-1.560175
5	-1.5	1.353240	1.35324050	1.353250
	0	-1.521243	-1.52123900	-1.521662
	1	-2.500135	-2.50013157	-2.500653
	3	-3.886555	-3.88655510	-3.886678
10	-1.5	2.200000	2.20002816	2.200456
	0	-2.257429	-2.25742372	-2.259142
	1	-3.660379	-3.66037218	-3.662782
	3	-5.635369	-5.62819631	-5.630445

**Table 3:** Numerical values of wall temperature gradient *θ’* (0)in the case of regular fluid (*Nb* = *Nt* = 10^-5^). Paranthesis show the corresponding results of Liu et al. [23].

*λ*	Pr	*θ’* (0)		
		*A = −2A*	*A = 0*	*A = 5*
0.0	0.7	0.6235675	-0.42582871	-1.641474
		(0.62361839)	(-0.42583804)	(-1.64165922)
	7	5.9319133	-1.8474565	-5.8975891
		(5.94094442)	(-1.84660569)	(-5.89780378)
0.5	0.7	0.76367407	-0.52152683	-2.0102735
		(0.76378454)	(-0.52154103)	(-2.01061361)
	7	7.2596204	-2.2631841	-7.2229124
		(7.27614126)	(-2.26162085)	(-7.22330493)
1.0	0.7	0.88177213	-0.6022019	-2.3211331
		(0.88194314)	(-0.60222359)	(-2.32165661)
	7	8.3764364	-2.6139021	-8.3401528
		(8.40176423)	(-2.61149481)	(-8.34075409)

**Fig 4:**
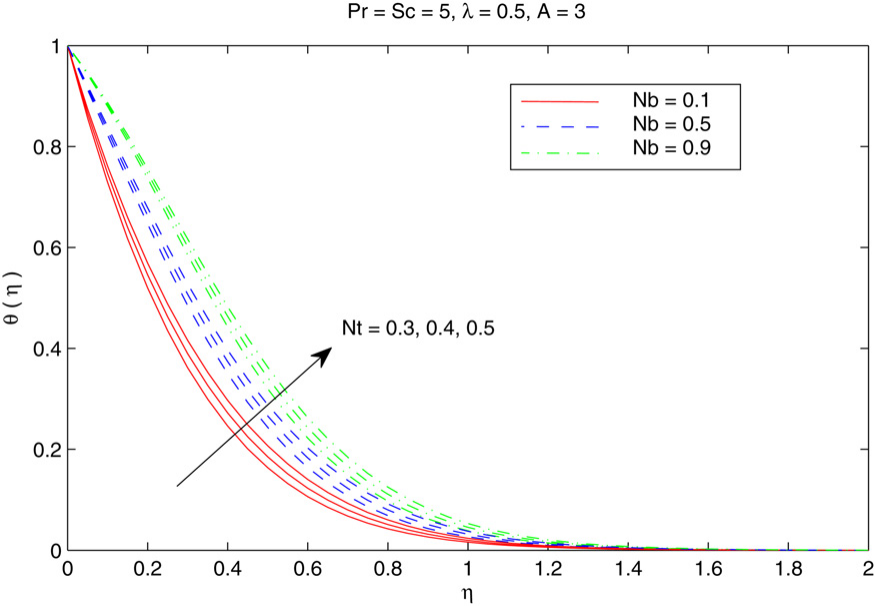
Effect of *Nb* and *Nt* on *θ*.

**Fig 5:**
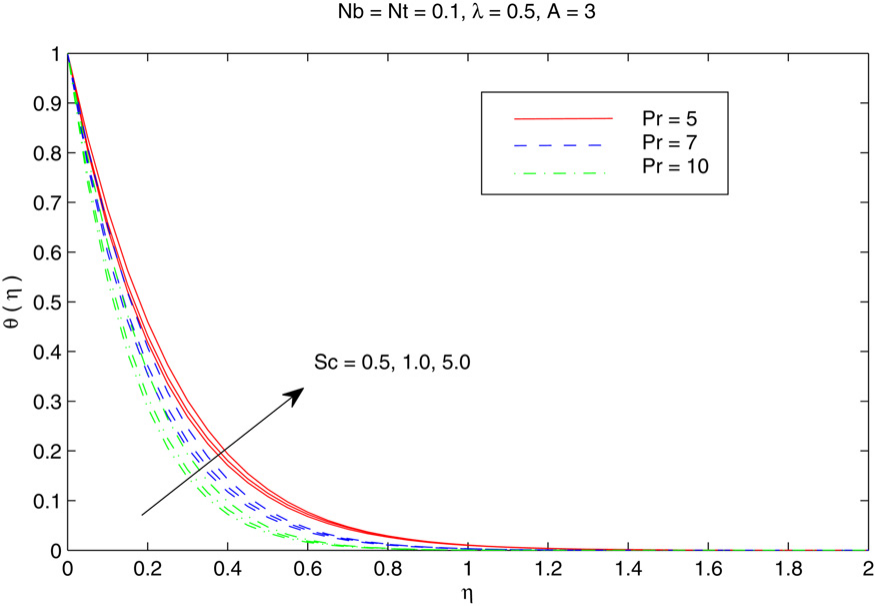
Effect of *Pr* and *Sc on* *θ*.

**Fig 6:**
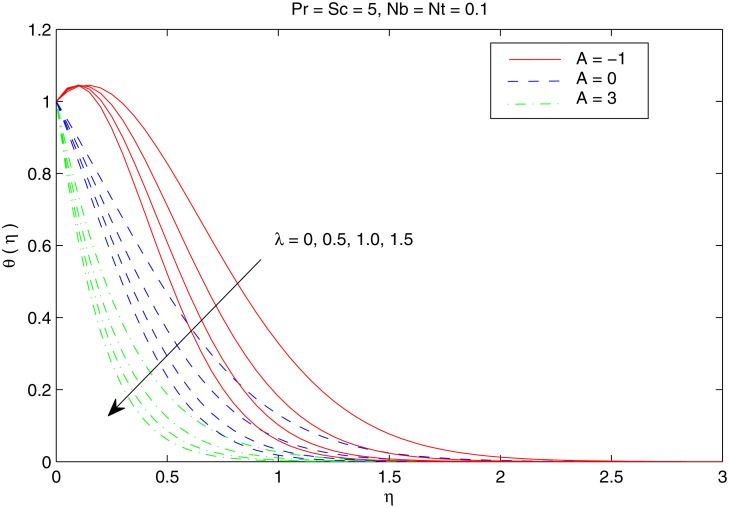
Effect of *A* and *λ* on *θ*.

Fig. 4 shows the variation in temperature distribution with an increase in parameters *Nb* and *Nt*. Brownian motion is the random motion of small colloidal particles suspended in a fluid, caused by the collision of the fluid molecules with the particles. For thermophoretic effect the motion of particles occurs due to the temperature gradient towards a cold surface and away from a hot one. An increase in the Brownian motion effect yields significant movement of nanoparticles which gives rise to the fluid kinetic energy and hence temperature increases. The difference in the temperature θ with *Nb* is similar for any considered value of *Nt*. Thermal boundary layer thickens when both *Nb* and *Nt* are simultaneously increased. Fig. 5 perceives the effects of *Pr* and *Sc* on temperature θ. For small Prandtl number fluids such as electrolyte solution the thermal conduction is dominant compared to convection. However, in high Prandtl number fluids such as water, ethylene glycol, engine oil etc. the convectionis effective in transferring energy from the sheet, compared to pure conduction. It may be noted here that values of Pr between 6 and 13 are for Al_2_O_3_ /water nanofluid (seeMaïga et al. [62] for details). Schmidt number is the analog of Prandtl number for mass transfer. It is observed that increasing values of Prcorresponds to weaker thermal diffusivity and thinner thermal boundary layer. This reduction accompanies with the bigger rate of heat transfer at the sheet. It is also noticed that temperature distribution only deviates near the stretching sheet when *Sc* is increased. Fig. 6 depicts the influence of velocity ratio *λ* on the temperature distribution for different values of temperature exponent parameter *A*. We notice that temperature *θ* decreases with an increase in *λ* for any considered value of *A*. In contrast to the problem of regular fluid [23], *A* = −1*A* does not correspond to the adiabatic case (which indicates no heat transfer between the sheet and the fluid) due to the presence of two additional effects in the energy equation. In fact, for *A* = −1, the profiles exhibit a reverse heat flow near the stretching sheet by forming “Sparrow—Gregg-type hill” *(SGH)*.

**Fig 7:**
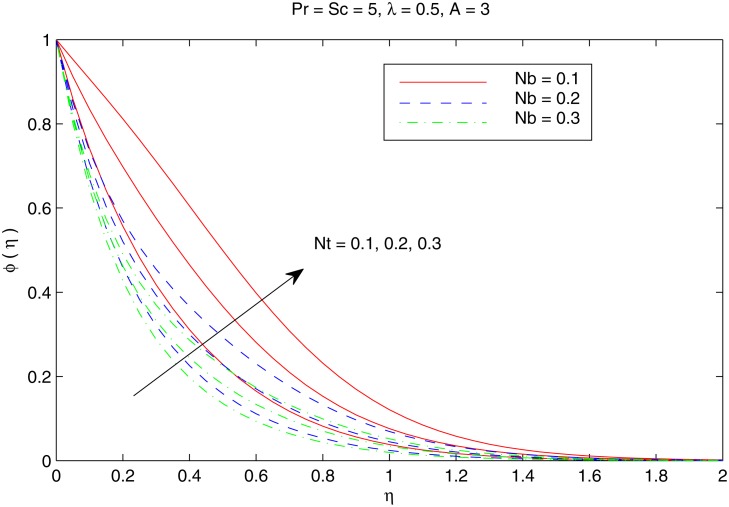
Effect of *Nb* and *Nt* on *ϕ*.

**Fig 8:**
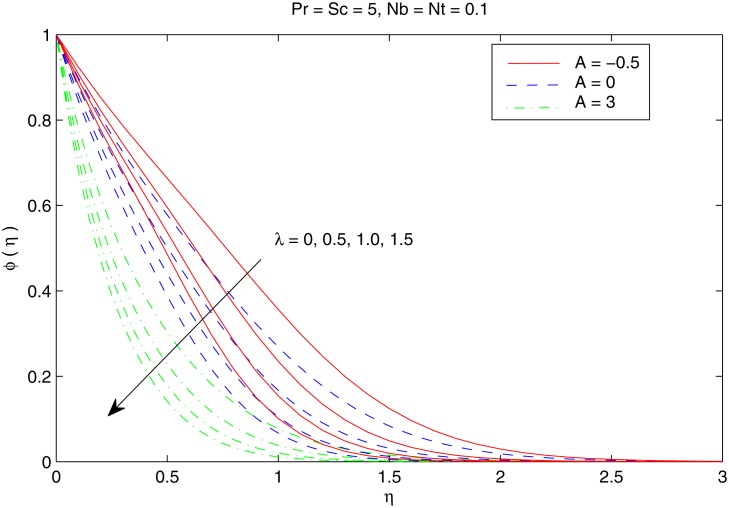
Effect of *A* and *λ* on *ϕ*.

**Fig 9:**
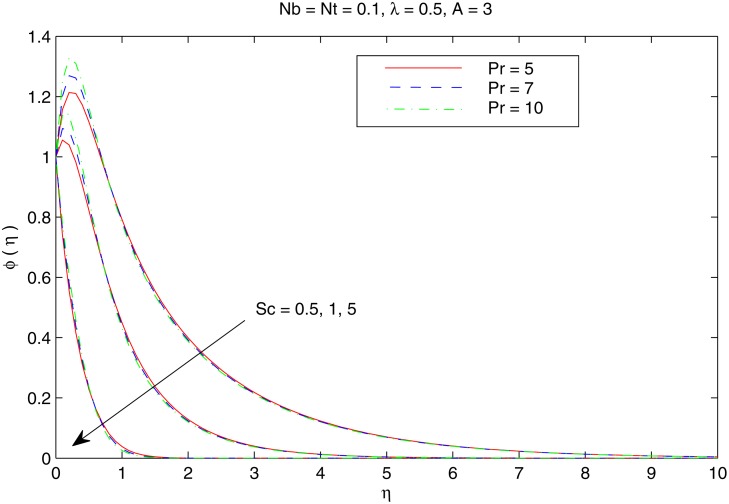
Effect of *Pr* and *Sc* on *ϕ*.

Fig. 7 elucidates the Brownian motion and thermophoresis effects on the nanoparticle volume fraction *ϕ*. Increasing values of *Nt* indicatesstronger thermophoretic force(due to temperature gradient) which shifts the nanoparticles from the hot sheet to the quiescent fluid thereby increasing the nanoparticle volume fraction boundary layer. Interestingly the increase in *ϕ* with *Nt* reduces when the Brownian motion effect intensifies i.e when *Nb* changes from 0.1 to 0.3. Fig. 8 shows the simultaneous effects of velocity ratio *λ* and temperature exponent *A* on the nanoparticle volume fraction boundary layer. Irrespective of the chosen value of *A*, *ϕ* increases as we move from unidirectional stretching sheet problem to the bidirectional one. Further volume fraction *ϕ* is a decreasing function of *A*. In other words an increase in the temperature and concentration distributions across the sheet results in the less penetration depth for *ϕ*. Fig. 9 shows the nanoparticle fractiondistribution *ϕ* for different values of *Sc* and *Pr*. It is found that bigger values of *Sc* indicates a weaker Brownian diffusion coefficient *D*_*B*_ and hence it corresponds to thinner concentration boundary layer. The profiles indicate the occurrence of SGH (even for positive values of *A*)when the Prandtl number is sufficiently large *Pr ≥ 5*. A minor decrease in *ϕ* with an augmentation in *Pr* is found just away from the stretching surface.

**Fig 10:**
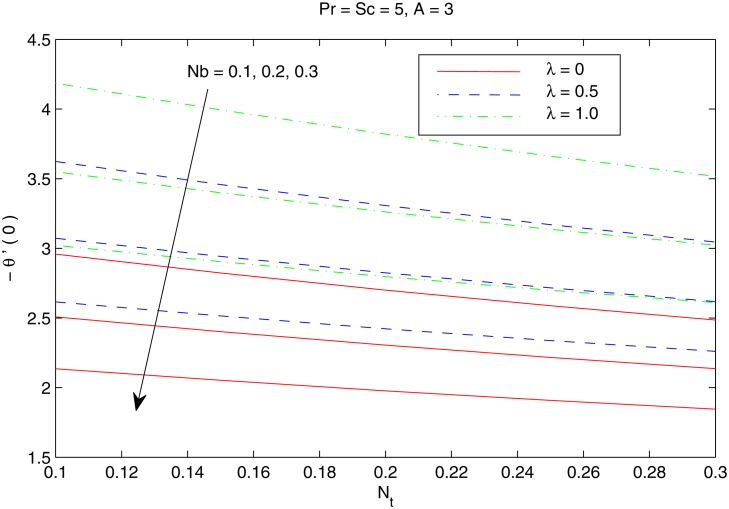
Effect of *λ*, *Nb* and *Nt* on *Nur*.

**Fig 11:**
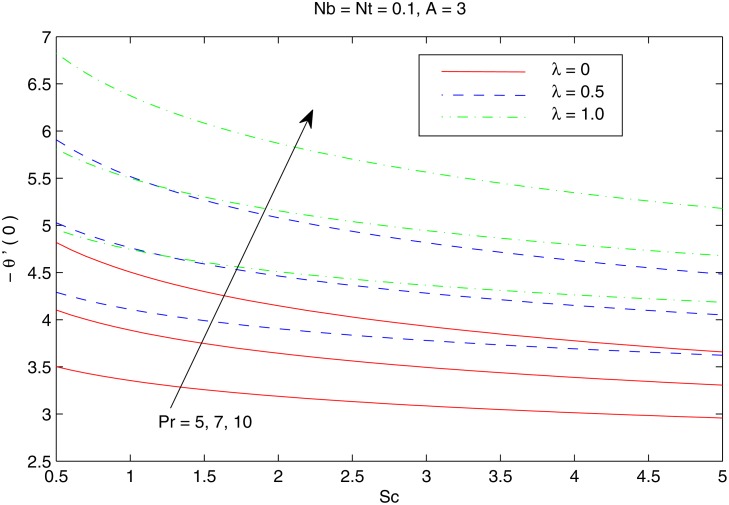
Effect of *λ*, *Pr* and *Sc* on *Nur*.

**Fig 12:**
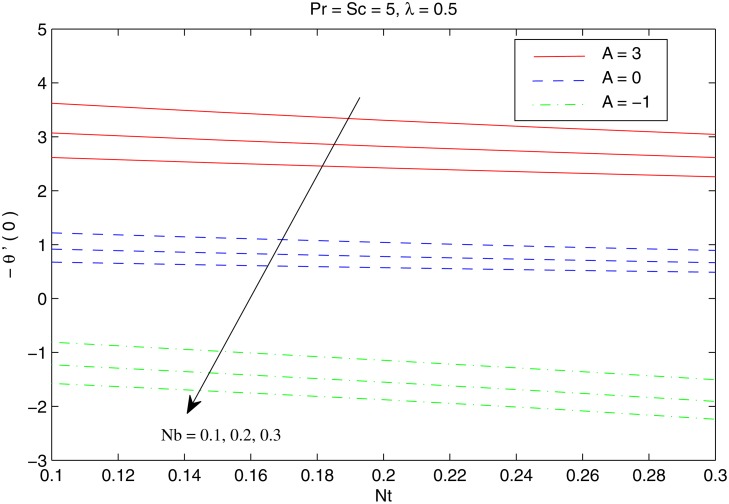
Effect of *A*, *Nb* and *Nt* on *Nur*.

**Fig 13:**
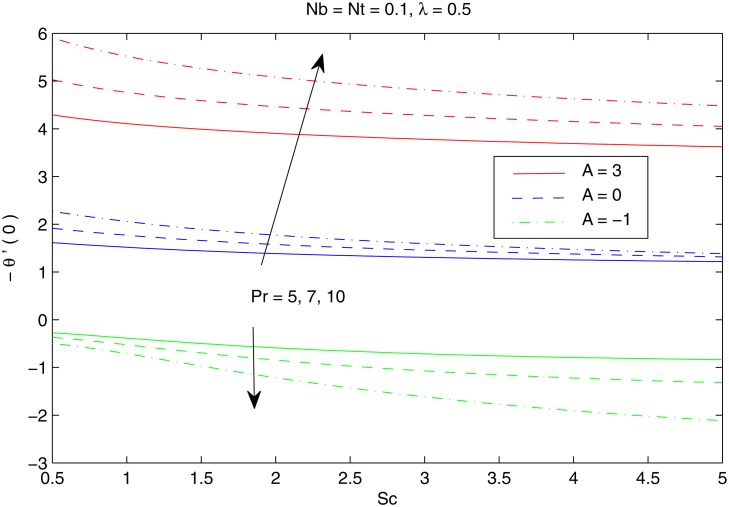
Effect of *A*, *Pr* and *Sc* on *Nur*.

**Fig 14:**
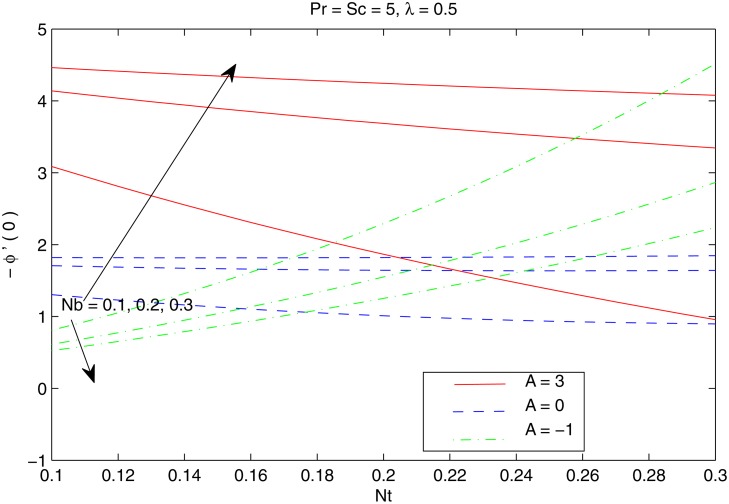
Effect of *A*, *Nb* and *Nt* on *Shr*.

**Fig 15:**
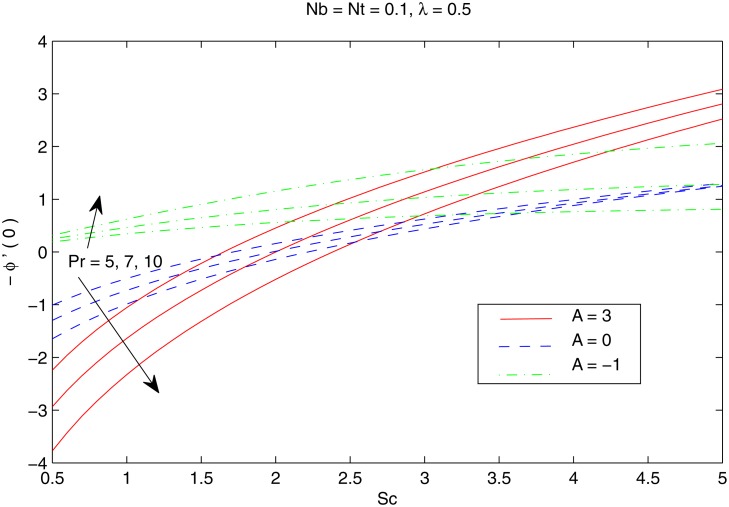
Effect of *A*, *Pr* and *Sc* on *Shr*.

The combined behavior of Brownian motion and thermophoresis parameters on the reduced Nusselt number *Nur* can be described from the Fig. 10. For some fixed value of *λ*, the surface heat transfer rate (|θ′(0)|) decreases with an enhancement of Brownian motion and thermophoresis effects. On the other hand |θ′(0)| is an increasing function of *λ* for any chosen value of *Nb* and *Nt*. Fig. 11 shows the variations in *Nur* with an increase in *Pr* and *Sc*. Increasing values of *Pr* corresponds to an increase in the wall slope of temperature function (earlier seen in Fig. 4) which eventually enhances the rate of heat transfer from the sheet. Moreover there is a decrease in *Nur* with an increase in *Sc* and this reduction is negligible for sufficiently large Schmidtnumber *(Sc ≥ 5)*. Figs. 12 and 13 plot the data given in Figs. 10 and 11 for different values of temperature exponent *A* by keeping *λ* fixed. Here the reduced Nusselt number is negative for *A* = −1 which is an indicator of the heat flow from the fluid to the sheet (as also noticed from Fig. 6). Figs. 14 and 15 are sketched to perceive the effects of different parameters on reduced Sherwood number *Shr*. For *A ≥ 0*, bigger values of *Nt* corresponds to stronger thermophoretic force which drives the nanoparticles from the hot surface to the quiescent fluid thereby forming a nanoparticle free layer near the sheet. As a result *Shr* decreases with an increase in *Nur* nd this reduction becomes significant when the Brownian motion strengthens. On the other hand when *A* = −1 (i.e when there is reverse heat flow) the wall mass flux becomes directly proportional to *Nt* for any chosen value of *Nb*. It is quite obvious from Fig. 5 that reduced Sherwood number escalates when Sc is increased for positive values of *A* and opposite trend is noticed for *A* ≤ −1.

## Conclusions

A numerical study is presented for the three-dimensional flow of nanofluid driven bya bi-directional exponentially stretching sheet. The temperature and nanoparticle concentration at the sheet are also exponentially distributed. The solutions are computed by an implicit finite difference scheme known as Keller-box method. The important points of this work may be summarized as follows:

1. The velocity increases in the *y-*direction and decreases in the *x*-direction when velocity ratio *λ* is increased. The entrainment velocity (*f*(*∞*)+g(*∞*))is an increasing function of *λ*.
2. For some negative values of temperature exponent *A*, the profiles reveal “Sparrow—Gregg-type hill” phenomenon. As a consequence, when *A* = −1, the behavior of parameters on the wall temperature gradient is opposite to that accounted for *A* ≥ 0.
3. Temperature *θ* increases when both Brownian motion and thermophoresis parameters increase. However the rate of heat transfer from the sheet reduces when the strengths of Brownian motion and thermophoresis effects are increased.
4. Nanoparticle fraction *ϕ* increases and rate of mass transfer from the sheet decreases when *N*_*t*_ is increased.
